# When closeness is effortful: Teachers’ physiological activation undermines positive effects of their closeness on student emotions

**DOI:** 10.1111/bjep.12506

**Published:** 2022-05-06

**Authors:** Tim Mainhard, Monika H. Donker, Tamara van Gog

**Affiliations:** ^1^ Department of Education Leiden University Leiden the Netherlands; ^2^ Department of Education Utrecht University Utrecht the Netherlands; ^3^ Department of Youth and Family Utrecht University Utrecht the Netherlands

**Keywords:** heart rate, physiological effort, student emotions, teacher closeness, teacher emotional labour

## Abstract

**Background:**

Student perceptions of teachers’ interpersonal closeness positively affect their emotions. If closeness is, however, effortful for the teacher (i.e., emotional labour, signalling less genuine closeness), this may undermine these positive effects. We tested this assumption by using student reports and external observations of teacher closeness and ambulant measures of teacher heart rate, to gauge teachers’ physiological effort connected to being close during class.

**Aims:**

We investigated the association between teachers’ physiological effort connected to closeness and students’ lesson‐focused emotions.

**Sample:**

75 teachers and their students (*N* = 1645) participated during one real‐life lesson.

**Methods:**

Teacher interpersonal closeness was continuously coded based on a video recording and teachers’ heart rate was measured continuously as an indicator of physiological effort. Students reported their emotions and perception of teacher closeness at the end of the lesson.

**Results:**

Multilevel models with student emotions as DVs and students’ perceptions of teacher warmth (L1 predictor) and teachers’ physiological effort when being close (i.e., an intra‐individual cross‐correlation, L2 predictor) were tested. As expected, students reported more positive and less negative emotions when they perceived more teacher closeness. The physiological effort connected to being close was not directly associated with student emotions; however, such effort moderated the effect of perceived closeness, especially with regard to negative student emotions (i.e., cross‐level interactions). The more effortful teacher closeness was, the less closeness protected students from negative emotions.

**Conclusions:**

In line with extant research on faking enjoyment and emotional labour, students seemed to be affected when teacher closeness was physiologically effortful, and overall positive effects of teacher closeness were undermined.

## INTRODUCTION

The importance of teacher interpersonal closeness, that is, the warmth and friendliness a teacher conveys during teaching, is rather unambiguous for student outcomes in general (Wentzel & Ramani, 2016) and for student emotions specifically (Lei et al., 2018). However, for some teachers showing behaviours related to closeness like being patient and supportive may be effortful, for example because they feel obliged to show such behaviour (Stark & Bettini, 2021). If showing interpersonal closeness during class is effortful, this could undermine positive effects of teacher closeness for student emotions. Therefore, in the current study, we expected that over and beyond average levels of teacher closeness that students perceive, the effort teachers put into showing closeness would also be associated with students’ lesson‐focused emotions. We tested this assumption by supplementing student reports of teacher closeness with classroom observations of teacher closeness and ambulant measures of teacher heart rate, to gauge teachers’ physiological effort connected to showing closeness during class.

### Teacher closeness and student emotions

In everyday life as well as in classrooms, the social environment is important for the emotions people experience (Baumeister & Leary, 1995; Martin & Dowson, 2009; Van Kleef & Côté, 2022). In classrooms, especially the way the teacher relates to students and behaves interpersonally is relevant for student emotions, as the teacher is the focal point of many social exchanges and teachers are responsible for the classroom process. In particular, closeness and the reinforcement of social bonds strengthen students’ positive affect and enjoyment, whereas anxiety and negative affect are related to damaged social bonds and exclusion. Closeness and belongingness with significant others can thus be understood as a basic human need and emotional reactions directly follow closeness in interpersonal relationships (Baumeister & Leary, 1995). Also in the classroom, student perceptions of teacher interpersonal closeness are associated with student emotions, as was indicated in a meta‐analysis of 65 studies (Lei et al., 2018), which reported a positive correlation of social teacher support with students’ positive affect (*r* = .34) and a negative correlation with negative affect (*r* = −.22).

Research focusing on teachers and their behaviour as antecedents of student emotions often focused on general affect measures rather than discrete student emotions such as enjoyment, boredom or anxiety (see the studies included in Lei et al., 2018). In line with Watson and Tellegen (1985), Pekrun's control value theory of achievement emotions (Pekrun's et al., 2006), however, advocates that studying discrete student emotions instead is important. This is because emotions with similar positive or negative affect, but different levels of activation, can have quite different functions in the school setting. For example, anxiety (a negative activating emotion) may lead students to actively avoid failure, whereas boredom (a negative deactivating emotion) may lead students to generally disengage from schoolwork. In addition, activating and deactivating emotions are usually the result of different classroom environments and teacher behaviour. Following Pekrun's work, we therefore focused on discrete student emotions rather than general affect as dependent variables in the present study.

In line with interpersonal theory, Mainhard et al. (2018) argued that a certain level of closeness is conveyed in essentially all behaviour a teacher shows in class, even if this is not explicitly intended or labelled as such (cf. Horowitz & Strack, 2010). For example, teacher enthusiasm and positive reinforcement of achievement (Becker et al., 2014; Frenzel et al., 2009; Goetz et al., 2013) as well as monitoring and clarity of instruction (Kunter et al., 2007) are all associated with higher levels of positive emotions in students. All of these teacher behaviours can be viewed as conveying teacher closeness because they indicate the teacher's concern with the students’ well‐being and positive development. Unpleasant emotions, such as anxiety, occur when students perceive teachers as cold or distant, such as punishing (Frenzel et al., 2007) or enforcing achievement (Goetz et al., 2006). Mainhard et al. (2018) found that student perceptions of teacher closeness accounted for substantial variance in student enjoyment and anxiety and findings by Sun et al. (2022) and Donker et al. (2021) generally corroborated these results. In line with this, Goetz et al. (2021) found in a longitudinal study that teacher closeness was associated with students’ positive emotional experiences (and negatively with unpleasant emotions). Interestingly, lower closeness levels were especially related to later *negative* emotions. Further, the associations between teacher closeness and students’ emotional experiences differed significantly across emotions. Thus, the hypothesized association between teacher closeness and students’ emotions may differ in strength depending on the specific emotion, also between two more pleasant emotions such as enjoyment or pride (see for example Goetz et al., 2010, 2021).

### Teacher closeness, display rules and physiological effort

Building social relationships can be effortful when people feel pressured or obligated to do so. Especially for professions with high levels of interpersonal contact, such as teaching, there has been increasing attention to the role of display rules in the degree of effort and stress one may experience (e.g., Chang, 2020). Display rules reflect occupational norms about what interpersonal behaviours are (not) acceptable in the context of one's work (Rafaeli & Sutton, 1989). Regarding teachers, work on display rules and underlying stress has mostly focussed on internal emotional process (i.e., emotional labour); however, emotions and interpersonal behaviour such as showing closeness are intertwined (Van Kleef & Côté, 2022) and (non‐) verbal behaviour can be viewed as the vehicle for communicating internal emotional states during interaction (Parkinson et al., 2005). For example, behavioural expressions of happiness or enjoyment signal general satisfaction with how the interaction is going (Van Kleef & Côté, 2022) and feed into the interaction partner's perceptions of closeness (Mainhard et al., 2018).

Chang (2020) showed that display rules can induce effortful expressive suppression of teachers’ negative emotions and of accompanying negative interpersonal behaviours, such as uncertain behaviour in case of negative emotions such as anxiety, or confrontational behaviour in case of anger—which all signal low closeness. However, display rules may not only function as a restriction or negative constraint for teachers. In a recent review, Stark and Bettini (2021) argued that for teachers, display rules can also function as a mandate to use behaviours signalling teacher closeness strategically (e.g., being generally friendly and engaging in positive interactions with students), mainly to provide emotional support and a positive classroom climate and to engage students in instructional content.

Nonetheless, the more effort teachers need to invest to comply with display rules, the more likely it is that teachers experience stress and feelings of burnout (i.e., emotional exhaustion, depersonalization and reduced personal accomplishment; Xanthopoulou et al., 2018). Along these lines, Donker et al. (2020) indicated, in a study using teacher heart rate as a measure of psychological effort, that the stronger associations between heart rate and externally observed teacher closeness were, the more dissatisfied teachers were about their teaching and the more anger they experienced during a lesson. Using heart rate, or physiological arousal in general, as an indicator of someone's momentary psychological effort is rather established in the fields of psychophysiology (Blascovich, 2008) and occupational psychology. For example, Rohrmann et al. (2011) showed that stricter adherence to work‐related display rules went together with an increased heart rate and higher stress levels.

The effort a teacher puts into showing closeness towards their students may thus be an important factor for classroom processes and for student emotions, as students may be negatively affected when (positive) teacher behaviour is effortful. On the one hand, actively re‐appraising the current classroom situation may be effortful (e.g., ‘this student is not actually boycotting my lesson, he is just tired’). On the other hand, actively suppressing emotions is effortful too. In more extreme cases, expressed behaviour may even become disconnected from what a teacher feels, like displaying closeness while in fact being angry. Gross (1998) and Taxer and Frenzel (2015) have labelled such more extreme cases of suppression as faking or inauthentic behaviour. Indeed, Keller et al. (2018) showed that inauthentic teacher enthusiasm went together with more negative and less positive student emotions, whereas with more authentic positive teacher behaviour, students reported higher levels of enjoyment and lower boredom. In sum, we expected that teachers’ effort connected to being close would be negatively connected with student emotions. In the current study, we used behavioural observations of teacher closeness during teaching and momentarily assessed teachers’ heart rate to estimate this effort.

### Heart rate as a measure of teacher stress and effort

Although the heart first and foremost provides the body with the oxygen needed to take action (Kreibig, 2010), physiological measures are increasingly being used as indicators of psychological phenomena, because of their objective, non‐invasive and continuous nature (Cacioppo et al., 2017; Ebner‐Priemer & Kubiak, 2007). Psychological constructs, such as effort, can be inferred by regressing out the heart rate changes due to teachers’ physical activity, resulting in an Additional Heart Rate measure, representing, as Myrtek (2004) framed it, emotional workload (also see Donker et al., 2018). This is also reflected in Blascovich’s (2008) biopsychosocial model, which describes how appraisals of goal incongruency and relevance of the task at hand can lead to an increased heart rate. According to this model, an increased heart rate is a general indicator of importance, urgency or task engagement which only occurs in motivated performance situation (Scheepers et al., 2012; Scholl et al., 2018). Heart rate has also more directly been associated with both situational demand and effort (Johnston et al., 2015).

The main advantage of using heart rate over other physiological measures (like skin conductance or blood pressure) is that heart rate can be measured continuously and reliably in ambulatory settings (Van Dijk et al., 2013). In addition, prolonged increased heart rate during work has been related to negative health outcomes in the long term (Vrijkotte et al., 2000).

In sum, taking heart rate into account may help to get insight into teachers’ momentary psychological effort connected to behaviours indicating closeness. Given Donker et al.’s (2020) earlier finding that a stronger positive connection between heart rate and closeness tends to go together with teacher anger, we expected that such effort has a generally negative connotation and is more likely to indicate emotional labour (i.e., putting effort into one's emotional display and therefore interpersonal behaviour) rather than, for example, enthusiasm.

### The current study

The goal of the current study was to further examine the link between teacher interpersonal closeness and students’ lesson‐focused emotions. Specifically, we expected that above being perceived as close, teachers’ physiological effort connected to being close would affect students’ emotions. To complement earlier studies, which primarily used student reports or self‐report of teachers’ overall closeness, we also used classroom observations of closeness. Instead of general student affect (e.g., compare the studies included in Lei et al., 2018), we assessed several discrete student emotions. Further, we used heart rate to tap into teachers’ effort from moment to moment during teaching. In line with Keller and Becker (2021), we expected that especially such momentary processes would be relevant for student emotions. Our main research question was: *To what degree does the physiological effort connected to teachers’ displays of closeness predict students’ lesson*‐*focused emotions beyond their general perceptions of teacher closeness?*

Next to testing the direct effect of teachers’ effort connected to their closeness, we also investigated: *T* *o what degree does* *physiological* *effort undermine positive effects of generally perceived closeness on the emotions that students experience?* Given that a lack of authenticity was associated with less positive student emotions (Keller & Becker, 2021) and that actively changing ones emotional display requires effort (Rohrmann et al., 2011; Taxer & Frenzel, 2015), we expected that the more effort is connected to showing close behaviour, the less helpful teacher closeness would be to enhance positive student emotions (i.e., a negative moderation effect) and the less students’ perceptions of closeness would protect them from negative emotions (i.e., a positive moderation effect).

## METHOD

### Participants

The data were collected during the Dynamics of Emotional Processes in Teachers (DEPTh; Donker, 2020) project. Participants were Dutch secondary school teachers, teaching a variety of subjects. The initial sample consisted of 80 teachers (41 females) with a mean age of 43.7 years (SD = 11.5 years) and on average 13.4 years teaching experience (SD = 9.7 years). Due to technical problems, no behavioural or heart rate data were available for 5 teachers, resulting in a sample of 75 teachers. For these teachers, time series data were available for on average 41 min 43 s (SD = 13 min 12 s). Teachers participated with one of their classes. In total, 1645 students with a mean age of 15.1 years (SD = 1.1 years) were included in the current sample. 50.3% of them were female. Average class size was 22 students (SD = 5). Most classes were taught by the participating teacher two or three times a week (73.8%; range 1 to 7 times) with a typical duration of 45 to 50 minutes per lesson (76.3%; range 45 to 90 min).

### Procedure

Teachers were sampled for convenience. The local ethics committee approved the study. Participation was voluntary and all teachers and students who participated in the present study signed an informed consent form. Of the students, 3.1% did not consent and were thus not included in the analyses.

Teachers were observed during one lesson. They were instructed to proceed normally and, if possible, to use activities including interpersonal interaction. The last ten minutes of the class were reserved for completing a paper questionnaire that recorded teachers’ and students’ perceptions of teachers’ interpersonal behaviour during the class period, as well as their positive and negative emotions. After participation, teachers received a gift certificate and a personal report with the summarized class results on the questionnaire items.

### Instruments

#### Teacher closeness

Interpersonal theory states that people's behaviour in interaction with others can be described by two dimensions in a circumplex structure (Fabrigar et al., 1997; Wubbels et al., 1985), which describe a person's dominance and closeness. The meta‐labels suggested by interpersonal theorists are agency and communion (Horowitz & Strack, 2010). The instruments used here are based on Interpersonal Theory and for the current study, we focused solely on closeness.

##### Student perceptions

We used the Questionnaire on Teacher Interaction (QTI; Wubbels et al., 1985) to assess students’ perceptions of teacher closeness. We used the 24‐item version with a five‐point Likert scale ranging from 1 (never) to 5 (always). Typical items assessing closeness include ‘this teacher is friendly’ or ‘this teacher is patient’. Reliability at the student level was α = .89 and α = .95 at the class level (ICC2, also see Donker et al., 2021).

##### External observation

The DEPTh data include codings of moment‐to‐moment interpersonal teacher behaviour using Continuous Assessment of Interpersonal Dynamics (CAID; Sadler et al., 2009). CAID uses the dominance or social influence and general sociability or closeness dimensions of the interpersonal circumplex as an underlying coding scheme, in line with the QTI. Coders continuously rated each change in teacher dominance and closeness by moving a joystick over the interpersonal circumplex. Computer software recorded these movements twice per second in terms of x‐ and y‐coordinates on a scale from −1000 to 1000. For the current paper, we only analysed the teacher closeness time series. Higher levels of closeness were coded when teachers were friendly, patient and supporting, both during plenary instruction as well as during dyadic interaction with a specific student. The deviation from the centre of the interpersonal circumplex indicated the intensity of the behaviour.

For the current study, the video recordings were rated by three trained coders. Segments with low reliability were recoded by the most deviant coder or (when none of the coders clearly deviated) by a fourth independent coder (less than 10% of all segments). The codes from the three coders with the highest reliability were averaged into one time series. We used 5 second aggregates of the coordinates in our analyses to match the ambulatory heart rate measures (see below). The ICC for these codings (two‐way random effects, consistency, three coders: Koo & Li, 2016) was .63 (SD = .13), indicating moderate agreement (LeBreton & Senter, 2007). For more information on the coding procedure and training, see Donker et al. (2020) and Lizdek et al. (2012).

#### Teacher heart rate

Teacher's heart rate was continuously monitored during class using the VU University ‐ Ambulatory Monitoring System (VU‐AMS; Willemsen et al., 1996). Seven electrodes were placed on the teachers’ chest before the lesson to measure the impedance cardiogram (ICG), electrocardiogram (ECG) and physical activity. The recording was started before the students entered the classroom and synchronized with the video cameras (and thus the later ratings of teacher closeness) by pressing the marker button at the VU‐AMS device in front of the camera, so that this moment linked both data sources. During the lesson, teachers could walk around freely in the classroom while wearing the device on a belt at their waist. After the lesson, the data were automatically checked for outliers and artifacts with the VU‐AMS software. All proposed corrections were also manually checked (less than 1% of the data). The corrected heart rate signal was exported per five seconds, as a balance between using the richness of the data and minimizing the amount of noise (Gratton & Fabiani, 2017; Porges & Byrne, 1992). The heart rate value at each 5 second interval was controlled for physical activity during that interval using the Additional Heart Rate approach (Houtveen & De Geus, 2009; Myrtek, 2004; see Donker et al., 2018). A teacher‐specific regression formula was used, which filtered out the heart rate changes beyond those that could be expected based on the physical activity of the teacher. The resulting heart rate values were person‐mean‐centred deviations from teachers’ individual mean scores and can thus be considered an indicator of effort. We were not able to include a resting baseline, due to the nature of real‐life teaching (i.e., tight schedules, and potential anticipatory stress before teaching starts). However, the additional heart rate approach accounts for individual teacher mean‐level differences in heart rate by using an individual regression formula, and only analysing deviations from the person‐centred mean (cf. Myrtek, 2004).

#### Student emotions

Students’ emotions were measured using an adapted version of the Achievement Emotions Questionnaire (Pekrun et al., 2011). All items were lesson focussed and to make the items applicable to a wide range of real‐life classroom contexts, we formulated shorter and more general items. The items are listed in Appendix A. Each item was rated on a scale from 1 (disagree) to 5 (agree). Items were organized according to nine discrete academic emotions which, in line with Pekrun (2006, 2017), reflected emotions experienced during the lesson (anger, anxiety, boredom and enjoyment) and after the lesson (‘now the lesson is over…’, pride, relaxation, relief, disappointment, and shame). Note that relief, although in essence a positive emotion, had a negative connotation in our approach (e.g., ‘I am glad the lesson is over’). The Cronbach's alpha and ICC2 (*k* = 21) of the scales was respectively .81 and .67 for anger, .72 and .42 for anxiety, .80 and .87 for boredom, .86 and .84 for enjoyment, .76 and .67 for pride, .83 and .57 for relaxation, .71 and .83 for relief, .80 and .59 for disappointment, and finally .76 and .53 for shame. This indicated that some emotions were more shared between students of a classroom than others. A CFA with nine discrete academic emotions indicated a satisfactory fit, χ^2^(491) = 2552.80, *p* < .001, CFI = .924, RMSEA = .049, SRMR = .045.

### Data analysis

We calculated the intra‐individual cross‐correlation between the time series of a teacher's heart rate and a teacher's closeness during teaching (as coded with CAID) as a measure of teachers’ effort connected to closeness. This cross‐correlation value for each teacher indicates the degree to which and the way (i.e., positively or negatively) changes in teacher closeness were connected to changes in teachers’ heart rate. A positive cross‐correlation indicated that physiological effort was increased during episodes with an increased closeness and lower during episodes with lower closeness. A negative cross‐correlation indicated that effort tended to be lower during class episodes with increased closeness and higher during episodes with relatively lower closeness displays. In the figures and tables below, we continue to refer to this cross‐correlation as ‘effort’.

In a series of multilevel analyses with discrete student emotions as dependent variables, students’ interpersonal perceptions of teacher closeness and the cross‐correlation were used as predictors. Models were fitted with students at the lower Level 1 and with teachers/classes at the higher Level 2. Student‐perceived closeness was entered as a classroom aggregate at L2 (grand‐mean centred) and as a personal view on teachers’ closeness at L1 (classroom‐mean centred). Also, the cross‐correlation, representing effort connected to being close, was grand‐mean centred. Next to testing simple effects of effort connected to closeness, we tested cross‐level interactions between teachers’ effort connected to closeness and student perceptions of teacher closeness. All analyses were done with SPSS version 25.0.

## RESULTS

**FIGURE 1:**
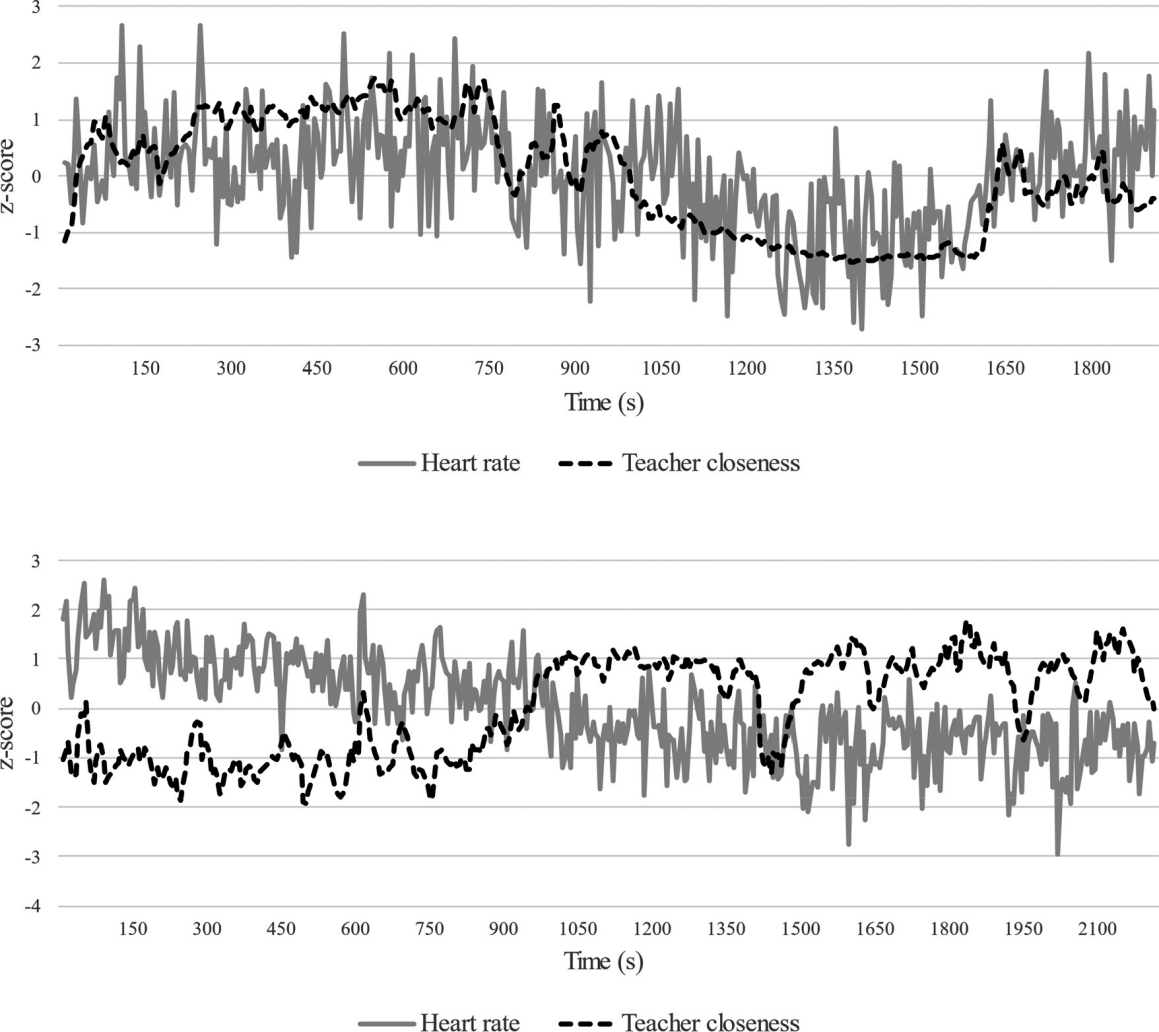
Two sets of time series selected from the current sample that present a positive cross‐correlation between Closeness and Heart Rate (*r* = .51; upper panel) and a negative cross‐correlation (*r* = −.63; lower panel)

**FIGURE 2:**
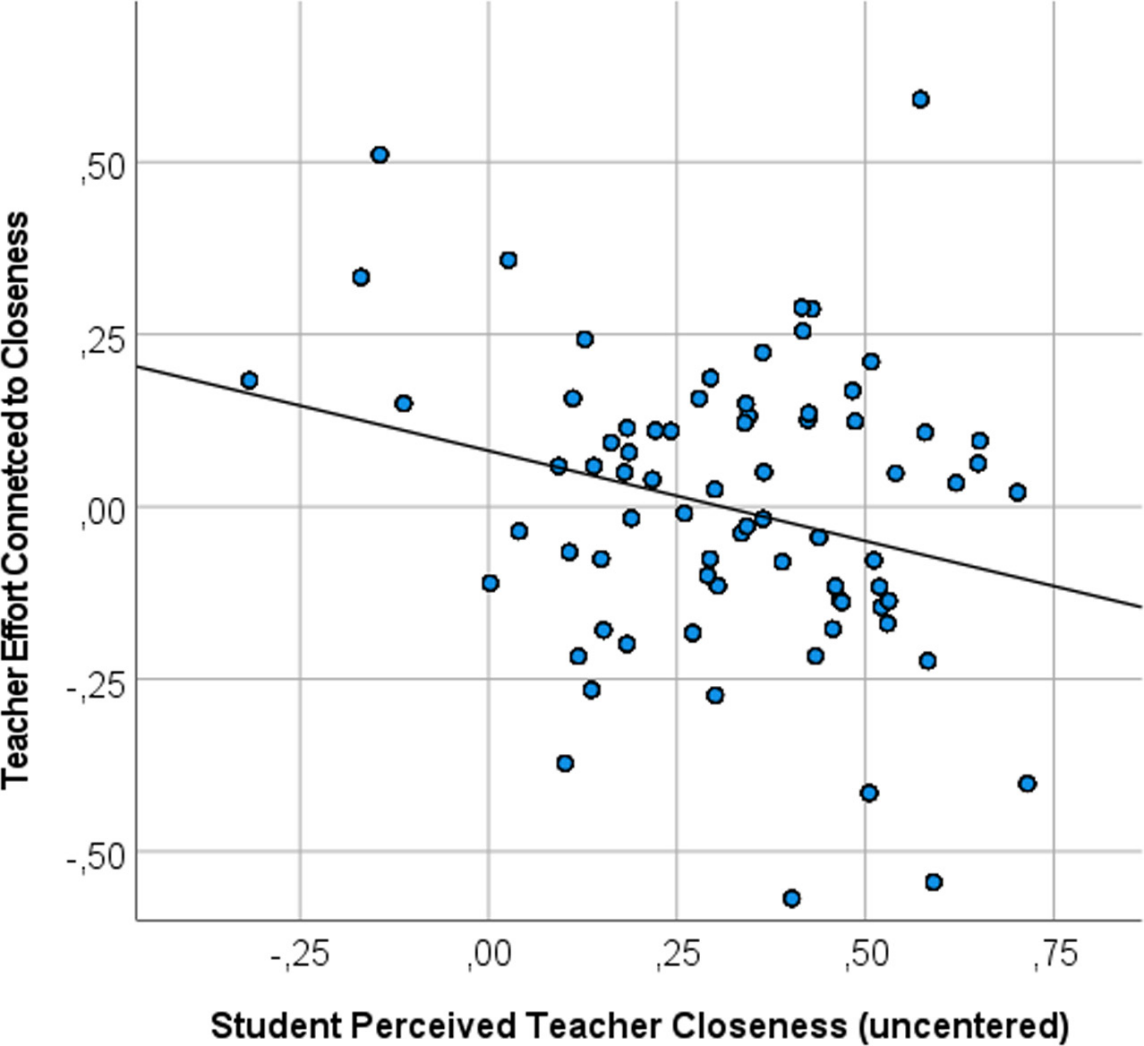
Teacher effort connected to closeness (observational data) plotted against general student perceptions of teacher closeness. *Note*. The correlation between student perceived teacher closeness (x‐axis) and teacher effort connected to being close (i.e., the cross‐correlation; y‐axis) was *r* = −.25. The y‐axis reflects the intra‐individual cross‐correlation between the time series of a teacher's heart rate and a teacher's closeness during teaching

Descriptives of the study variables are provided in Table 1. There was a large range in the cross‐correlation reflecting the degree to which teachers’ physiological effort (heart rate) was connected to showing closeness (see Table 1). To further illustrate this measure, Figure 1 depicts heart rate and closeness time series of two teachers that represent a positive (upper panel) and negative cross‐correlation (lower panel). Further, Figure 2 shows how effort during episodes of closeness was somewhat more or less likely for teachers who were perceived by students as generally more or less friendly or close.

**Table 1:** Descriptives.

	Mean	SD	Min	Max
Teacher Closeness (as perceived by students)^a^	.31	.29	−.88	.96
Teacher Effort Connected to Closeness (cross‐correlation)^b^	−.08	.21	−.65	.51
Student Enjoyment	3.10	0.98	1	5
Student Pride	3.62	0.90	1	5
Student Relaxation	3.97	0.80	1	5
Student Boredom	3.05	1.02	1	5
Student Anger	1.61	0.77	1	5
Student Anxiety	1.60	0.68	1	5
Student Relief	2.92	1.02	1	5
Student Disappointment	1.50	0.69	1	5
Student Shame	1.51	0.63	1	5

^a^ ICC1 = .45; ICC2 = .95, range −1 to 1.

^b^ This measure reflects the intra‐individual cross‐correlation between the time series of a teacher's heart rate and a teacher's closeness during teaching.

As can be seen in Figure 2, for teachers who were perceived as generally less close (at the left side of the graph), physiological effort tended to be more strongly and more positively connected to being close. For teachers who were perceived as moderately close (e.g., closeness = .25), effort was less strongly connected to their closeness during teaching.

In Table 2, an overview of the correlations between the study variables is provided. Both students’ perceived closeness and observed closeness were statistically significant and in the expected direction associated with student emotions. Teacher effort connected to being close was, however, unrelated to student emotions.

**Table 2:** Correlations of the study variables within (lower triangle) and between classrooms (upper triangle).

	1	2	3	4	5	6	7	8	9	10	11	12	13	14
1. Closeness (student report)		.66**	.32**	.75**	−.71**	−.78**	−.43**	−.73**	−.28*	−.32**	−.09	−.04	.44**	−.24*
2. Enjoyment	.38**		.36**	.69**	−.82**	−.57**	−.31**	−.80**	−.19	−.18	−.12	−.30	.23	−.07
3. Pride	.20**	.33**		.32**	−.53**	−.37**	−.29*	−.34**	−.52**	−.49**	−.15	−.10	.17	.06
4. Relaxation	.41**	.41**	.32**		−.68**	−.77**	−.63**	−.76**	−.42**	−.40**	−.01	.05	.35**	−.19
5. Boredom	−.40**	−.50**	−.31	−.23**		.73**	.34**	.87**	.30**	.33**	.13	.15	−.42**	.12
6. Anger	−.47**	−.28**	−.18**	−.44**	.36**		.60**	.72**	.51**	.51**	.09	.03	−.40**	.15
7. Anxiety	−.25**	−.09**	−.21**	−.43**	.18**	.52**		.40**	.65**	.66**	−.01	−.11	−.22	.19
8. Relief	−.33**	−.34**	−.10**	−.21**	.59**	.32**	.21**		.36**	.32**	.13	.07	−.34**	.12
9. Disappointment	−.20**	−.08**	−.34**	−.30**	−.30**	.37**	.53**	.22**		.82**	−.09	.05	−.16	.01
10. Shame	−.21**	−.07**	−.28**	−.25**	.21**	.37**	.48**	.23**	.72**		−.13	.10	−.27*	.02
11. Student Gender	−.01	−.08**	−.06*	−.07**	−.01	−.04	.01	−.06*	−.05*	−.10**			.02	−.04
12. Teacher heart rate													−.22	−.14
13. Closeness (observed)														−.33**
14. Effort^a^														

Note

Within classroom correlations are based on classroom mean centred scores (*N* = 1645 students); between classroom correlations are based on classroom aggregated scores (*N* = 75 classrooms). Variables 12 to 14 are classroom level constructs. Student gender was coded 0/1 with 1 for girls, the ratio between boys and girls was used at the class level.

^a^ Effort represents the intra‐individual teacher correlation between moment‐to‐moment observed closeness and teacher heart rate.

We also checked whether teacher experience and gender, as well as students’ age and gender were associated with the variables included in our study. For student gender, small correlations were found at the student level for some of the emotions (see Table 2). Girls tended to report somewhat lower enjoyment and pride (cf. Frenzel et al., 2007) as well as relief, disappointment and shame, which was not in line with Frenzel et al. (2007) and which may be explained due to the fact that our items were not test‐ or task‐focussed. Teacher gender and experience as well as class size and percentage of boys in class were unrelated to the study variables. There was no association found between teacher experience and teachers’ effort connected to being close; *r* = .17, *p* = .16, *N* = 75.

In Table 3, the final multilevel models are summarized per student emotion. The initial variance decomposition models (i.e., the ‘empty’ models) are provided in Appendix B. In general, when students perceived more teacher closeness (both shared with classmates at L2 and idiosyncratically at L1), students reported more positive and less negative emotions. In line with the bi‐variate correlations reported in Table 2, no direct effect of teacher physiological effort connected to closeness was found for any of the emotions included. However, physiological effort moderated the association between perceived teacher closeness and student emotions, especially with regard to negative student emotions (a cross‐level interaction, see Table 3).

**Table 3:** Multilevel models predicting student emotions with teacher closeness.

	Enjoyment	Pride	Relaxation	Boredom	Anger	Anxiety	Relief	Disappointment	Shame
Fixed effects									
Intercept	3.34 (.08)**	3.78 (.08)**	3.97 (.02)**	3.08 (.05)**	1.69 (.05)**	1.59 (.05)**	3.09 (.08)**	1.63 (.06)**	1.72 (.05)**
Closeness (gmc)	1.54 (.20)**	.55 (.18)**	1.12 (.12)**	−1.80 (.22)*	−1.19 (.11)*	−0.40 (.10)	−1.76 (.19)**	−0.33 (.13)*	−0.34 (.11)**
Effort^a^	0.22 (.20)	0.25 (.18)	−0.03 (.12)	−0.12 (.21)	−0.05 (.11)	0.08 (.10)	−0.15 (.19)	−0.08 (.13)	−0.07 (.11)
Student gender	−0.16 (.04)**	−0.11 (.04)*	−0.10 (.04)**	−0.03 (.04)	−0.06 (.03)	0.01 (.03)	−0.11 (.05)*	−0.08 (.03)*	−0.14 (.03)**
Closeness (cmc)	1.58 (.10)**	.84 (.10)**	1.46 (.09)**	−1.67 (.11)**	−1.53 (.09)**	−0.83 (.08)**	−1.45 (.11)**	−0.62 (.08)**	−0.64 (.08)**
Effort*Closeness	−0.60 (.47)	−0.89 (.49)	−0.82 (.44)	1.09 (.54)*	1.41 (.42)**	1.07 (.38)**	1.29 (.52)*	1.19 (.37)**	1.19 (.36)**
Random effects									
L2 (Intercept)	.09	.06	.02	.11	.02	.01	.08	.03	.02
Pseudo R2	52%	14%	71%	54%	71%	50%	60%	0%	0%
L1 (Residual)	.65	.71	.47	.68	.39	.41	.76	.42	.36
Pseudo R2	15%	7%	20%	17%	25%	9%	13%	9%	8%
Random slope	<.01	<.01	.09	.18	.13	.02	.05	<.01	.03

Note

Standard errors are within brackets. Effort represents the intra‐individual cross‐correlation between teacher heart rate and teacher closeness as externally observed. Effort*Closeness represents the cross‐level interaction between cluster mean centred student perception of closeness and the teacher level cross‐correlation between physiological effort and observed teacher closeness.

^*^p < .05; **p < .01.

^a^ Effort represents the intra‐individual teacher correlation between moment‐to‐moment observed closeness and teacher heart rate.

**FIGURE 3:**
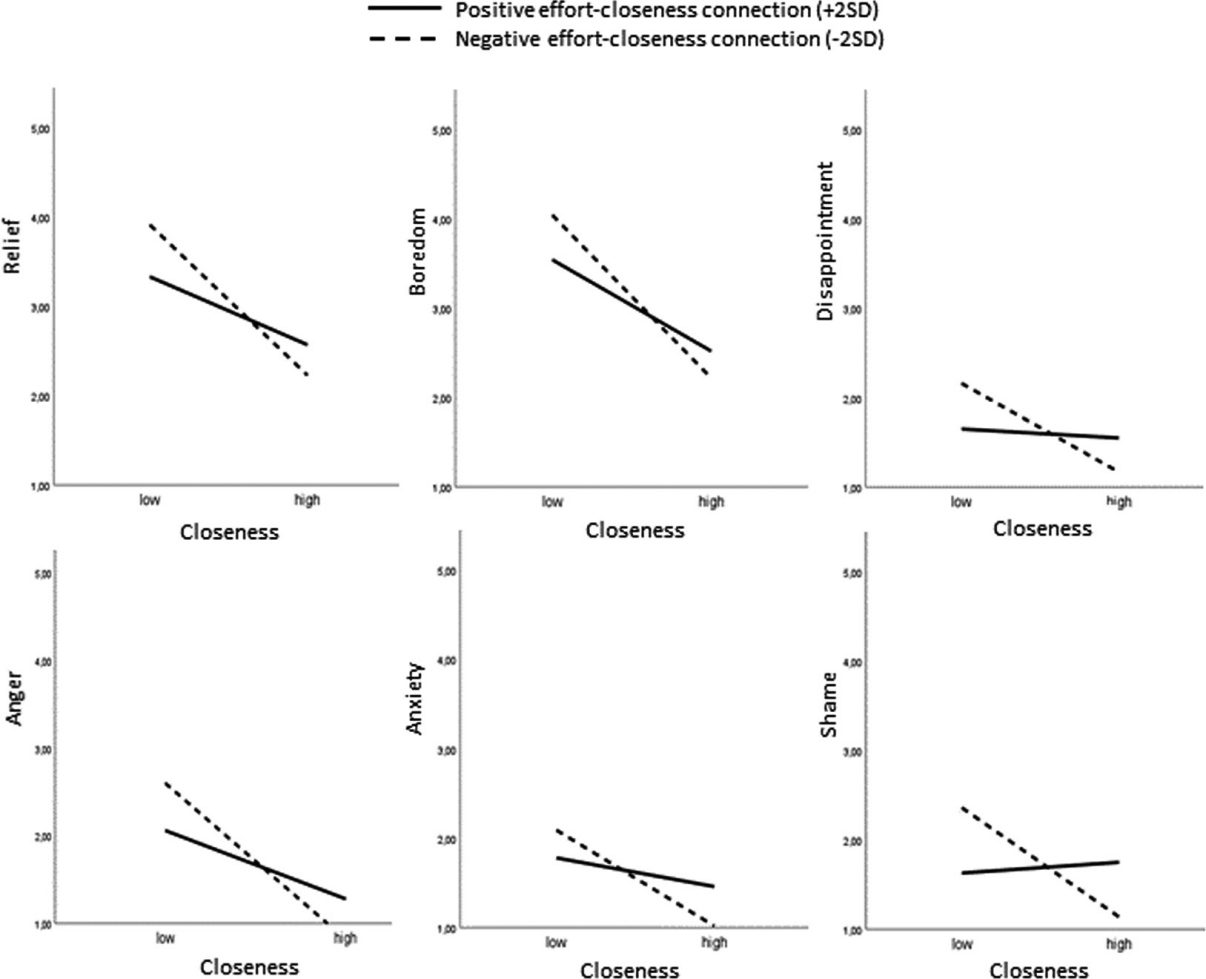
Moderation effects illustrated for student emotions and low and high perceived teacher closeness (± 2 SD) and for positive versus negative effort and closeness cross‐correlations (solid versus dotted lines). *Note*. The solid lines represent teachers who typically show greater physiological effort when displaying closeness in class, the dotted lines represent teachers with greater physiological effort when displaying unfriendly behaviour or low closeness

The graphs depicted in Figure 3 illustrate that, if a student generally perceived high levels of closeness, they also tended to report lower levels of negative emotions. If effort was positively connected to showing closeness in class, however (i.e., a positive cross‐correlation between physiological effort and observed closeness; solid line), perceived closeness protected students less well of negative emotions. That is, the positive effects of closeness were weaker. In contrast, if a teacher typically had an increased heart rate when showing negative behaviour (dotted line), negative effects of low closeness were predicted to be even stronger. Students of these teachers were predicted to report the most negative emotions in our sample.

## DISCUSSION

Positive effects of teachers’ interpersonal closeness on students are quite unambiguous (Wentzel & Ramani, 2016). However, if being close during class is effortful for teachers, this may counter such positive effects (Chang, 2020; Keller & Becker, 2021). We tested this assumption by using classroom observations of teacher closeness and ambulant measures of teacher heart rate during teaching, to gauge how effortful closeness was for teachers and to investigate associations with students’ emotions. The major finding was that increased effort with positive or close teacher behaviour indeed undermined positive effects of closeness, whereas increased effort during negative teacher behaviour amplified negative effects on student emotions. Physiological measures of effort thus helped us to tap into teachers’ personal, moment‐to‐moment, affective reactions coupled with their behaviours. Notably, no direct effect of teachers’ effort connected to showing closeness was found, but effort connected to closeness acted as a moderator.

### Effortful teacher closeness and student emotions

We confirmed that if students perceived more teacher closeness, they were more likely to report more positive and less negative lesson‐focussed emotions (cf. Goetz et al., 2021; Mainhard et al., 2018). However, the protective value of closeness, especially for negative student emotions, was undermined *if teachers experienced more physiological effort when displaying closeness* in class. This is in line with Goetz et al. (2021) where especially student boredom and anger showed reciprocal relationships with teacher closeness. One reason why the undermining effect of effortful closeness was present in particular for negative emotions, could be that negative rather than positive teacher emotions were involved with higher heart rates (Donker et al., 2020) and that these negative emotions were subsequently picked up by students and mirrored in their own emotions (Parkinson et al., 2005). Although Parkinson et al. (2005; also see Van Kleef & Côté, 2022) expect emotion transmission to occur via (non‐) verbal behaviour and facial expressions, students’ closeness perceptions as such were only moderately associated with effortful closeness. Thus, also other, more intricate pathways beyond perceptions of closeness, seemed to play a role.

The undermining effect of effortful closeness was especially apparent for students’ anxiety, disappointment and shame; for these emotions, the interaction effect meant that the already relatively small positive effects of teacher closeness were cancelled out completely (see Figure 3). Thus, closeness became irrelevant especially for negative emotions with an outcome focus rather than an activity focus (i.e., being bored or angry; Pekrun, 2017). Anxiety and shame are, moreover, connected to feelings of uncontrollability and avoidance of failure. It thus seems that especially the quality of being dependable as a teacher and as providing support for student school‐related activities (Wentzel & Ramani, 2016) was undermined if closeness was effortful. Given our results and those reported in Donker et al. (2020), who found that a stronger positive connection between heart rate and closeness tends to go together with teacher anger, we think it is fair to conclude that during teaching, effort connected to closeness has a generally negative connotation, rather than, for example, indicating teacher enthusiasm (cf. Kreibig, 2010).

Further, *if teachers’ heart rate typically increased when displaying low levels of closeness*, thus while being unfriendly or cold with students, negative effects on student emotions were even more pronounced. This could indicate that these teachers were more prone to ‘reactive aggression’, that is being irritable or hostile in response to student behaviour. In research outside the educational context, more sympathetic activation, such as an increased heart rate, has been related to reactive aggression in interpersonal settings (Murray‐Close et al., 2017; Naim et al., 2021). Moreover, in terms of emotion regulation, the combination of unfriendly behaviour and a heightened heart rate seems to imply the free expression of negative emotions rather than emotional labour, as reflected in reappraisal or the suppression of negative emotions (Chang, 2020).

### Emotional labour: Effortful or inauthentic closeness?

For some teachers, showing closeness went together with significantly more effort than for others. In line with extant research on faking enjoyment and emotional labour (Keller et al., 2018; Taxer & Frenzel, 2015), students seemed to be affected when teacher closeness was physiologically indicated as effortful and overall positive effects of teacher closeness were undermined.

As Chang (2020) and others have argued, this could be induced by display rules that lead to effortful expressive suppression of teachers’ negative emotions. Gross (1998) and Taxer and Frenzel (2015) have labelled more extreme cases of suppression as faking emotions and displays of inauthentic behaviour. In the current study, it is not possible to discern exactly which teachers actually faked behaviour or just put effort into being friendly. A heightened heart rate could either imply effortful suppression of emotions, but also a more healthy effortful reappraisal of negative classroom situations. The fact that effortful closeness was only moderately strong connected to students’ perceptions of closeness implies that putting effort into being a nice teacher is not detrimental per se to being perceived as close. Moreover, it is of course commendable if teachers try to achieve a positive interpersonal classroom climate. Indeed, Stark and Bettini (2021) showed that teachers can experience display rules rather as a mandate to use such friendly behaviours strategically in class to provide emotional support and to engage students.

### Implications for educational practice

Overall, effortful closeness seems to be undesirable for both students and teachers. Over‐emphasizing teacher closeness might in the long term not only negatively affect student emotions, but might also harm teachers’ own well‐being if too much effort is induced. Although some effort and short‐term feelings of stress are not problematic, it should be noted that pressuring for teacher closeness could cause longer term occupational stress (cf. Chang, 2020). Instead, discussing display rules more explicitly during teacher training, as well as training teachers how to use and internalize reappraisal could benefit both teachers and students (Chang, 2020; Gross, 1998). For example, so‐called deep acting, which includes reappraising the classroom situation in more positive ways (‘my students are not motivated because it is Friday afternoon, not because my lesson is boring’) can help to act in less effortful ways rather than simply suppressing one's anxiety or anger (also see Keller & Becker, 2021) or acting out one's negative emotions. Importantly, providing teachers with positive behaviour alternatives that can be used in a difficult classroom situation is likely to increase teacher efficacy and can help to reduce teachers’ stress connected to interpersonal classroom interaction (Friedman, 2003).

### Using physiological measures, limitations and future directions

On the one hand, a strength of the current study was that through the use of physiological measures, we were able to assess teachers’ effort in a rather objective way. In contrast to self‐report (cf. Keller & Becker, 2021; Taxer & Frenzel, 2018), our effort measure was not subjected to response or self‐report biases and tracking heart rate did not disrupt the ongoing lesson like, for example, experience sampling would have. On the other hand, it should be noted that first and foremost the heart provides the body with oxygen. Although there is a strong tradition of using heart rate to assess psychological effort (Blascovich, 2008; Kreibig, 2010; Myrtek, 2004), one should keep in mind that psychological effort, if based on heart rate, remains an inferred measure.

Further, a drawback of our approach is that we have only circumstantial evidence about what exactly induced a higher or lower heart rate. Other personal factors or classroom processes might have affected teacher's heart rate next to showing closeness. Future research could validate teachers’ emotional valence during, and their appraisals of, classroom situations with an increased heart rate. There is some evidence that classroom processes unrelated to interpersonal processes have physiological correlates as well, such as whole class instruction (Junker et al., 2021). It could be insightful to conduct video and physiology stimulated recall interviews with teachers and students. Also, correlating our effort measure with self‐report and student reports of (in‐)authenticity of teacher behaviour could help to better interpret our physiological measure. Overall, physiological measures seem to be especially informative if they are well embedded in other contextual measures (Houtveen & De Geus, 2009). In this regard, it should be noted that teachers’ heart rate can be expected to not only affect teachers themselves and their students, but that the classroom context and how students behave is also likely to be reflected in teachers’ physiology.

## CONCLUSION

Showing closeness in class is important for students’ emotions, but it can be effortful for teachers at the same time. The current investigation confirmed that the effort teachers put into being close can undermine positive effects of teacher closeness—specifically with regard to negative student emotions. On a more positive note, effortful or not, teacher closeness had a positive effect on almost all student emotions we assessed. This hopefully encourages teachers to invest in close and positive interactions with their students. So‐called deep acting, or internalized reappraisal of negatively perceived classroom processes, could ultimately help teachers to feel and show genuine closeness without much effort (Chang, 2020; Gross, 1998).

## CONFLICT OF INTEREST

All authors declare no conflict of interest.

## AUTHOR CONTRIBUTION

**Tim Mainhard:** Conceptualization; Formal analysis; Funding acquisition; Writing – original draft. **Monika H. Donker:** Conceptualization; Data curation; Writing – review & editing. **Tamara van Gog:** Supervision; Writing – review & editing.

## Data Availability

The data are available on request from the second author.

## Appendix A

### Lesson‐focused emotions questionnaire

Items were translated and adapted from the Achievement Emotions Questionnaire (Pekrun et al., 2011) and asked in a (quasi) randomized order. Answer options ranged from 1 (oneens [disagree]) to 5 (eens [agree]). Note that the English items listed here are merely translations of our Dutch items. The English items below were not psychometrically tested in the current study. The first part of the questionnaire asked how students felt during the lesson and the second part focused on students’ concurrent evaluative emotions.

Instruction: De volgende vragen gaan over hoe jij de afgelopen les vond. [The next questions are about how you experienced the past lesson.]

**Table:**

	Dutch (original)	English (translated)
	Tijdens deze les …	During this lesson …
Relaxation	… voelde ik me rustig	… I felt calm
	… voelde ik me relaxed	… I felt relaxed
	… voelde ik me op mijn gemak	… I felt comfortable
	… was ik ontspannen	… I was relaxed
Enjoyment	… had ik plezier	… I had fun
	… was ik enthousiast	… I was enthusiastic
	… heb ik genoten	… I enjoyed myself
	… vond ik het leuk	… I liked it
Boredom	… vond ik het saai	… I found it boring
	… verveelde ik me	… I was bored
	… dwaalden mijn gedachten af	… my thoughts drifted away
	… wilde ik dat de les voorbij was	… I wanted the class to be over
Anger	… voelde ik me boos	… I felt angry
	… was ik gefrustreerd	… I was frustrated
	… voelde ik irritatie	… I was irritated
	… ergerde ik me	… I was annoyed
Anxiety	… maakte ik me zorgen	… I was worried
	… was ik nerveus	… I was nervous
	… voelde ik me gespannen	… I felt tense
	… was ik bang iets verkeerd te doen	… I was afraid to make mistakes

Instruction: De volgende vragen gaan over hoe jij je op dit moment voelt als je terug kijkt op de afgelopen les. [The next questions are about how you feel right now, looking back on the past lesson.]

**Table:**

	Dutch (original)	English (translated)
	Als ik terugkijk op de afgelopen les …	When I think of the past lesson …
Relief	… ben ik blij dat deze les afgelopen is	… I am glad the lesson is over
	… voel ik me beter dan tijdens de les	… I feel better than during the lesson
	… voel ik opluchting dat het voorbij is	… I am relieved that it is over
Pride	… vind ik dat ik het goed heb gedaan	… I think I did a good job
	… ben ik tevreden over mezelf	… I am content with myself
	… ben ik trots op mezelf	… I am proud of myself
Disappointment	… ben ik teleurgesteld in mezelf	… I am disappointed in myself
	… valt het me tegen hoe ik het heb gedaan	… I am disappointed with how with how I performed
	… voelt het alsof ik gefaald heb	… I feel like I failed
	… voel ik me terneergeslagen	… I feel down
Shame	… heb ik een schuldgevoel	… I feel guilty
	… schaam ik mij	… I am ashamed
	… had ik de les anders willen doen	… I would have liked to have done things differently
	… heb ik spijt van dingen die ik heb gedaan	… I regret some of the things I did

## Appendix B

### Variance decomposition of student emotions

**Table:**

	Enj	Pride	Relax	Bored	Anger	Anx	Relief	Disap	Shame
Fixed effects									
Intercept	3.12 (.06)	3.62 (.04)	3.97 (.04)	3.02 (.05)	1.60 (.04)	1.60 (.02)	2.90 (.06)	1.51 (.03)	1.51 (.02)
Random effects									
L2 (Intercept)	.19	.07	.07	.24	.07	.02	.20	.03	.02
L1 (Residual)	.76	.76	.59	.82	.52	.45	.87	.46	.39

## References

[bjep12506-bib-0001] Baumeister, R. F. , & Leary, M. R. (1995). The need to belong: Desire for interpersonal attachments as a fundamental human motivation. Psychological Bulletin, 117(3), 497–529. 10.1037/0033-2909.117.3.497 7777651

[bjep12506-bib-0002] Becker, E. S. , Goetz, T. , Morger, V. , & Ranellucci, J. (2014). The importance of teachers’ emotions and instructional behavior for their students’ emotions–An experience sampling analysis. Teaching and Teacher Education, 43, 15–26. 10.1016/j.tate.2014.05.002

[bjep12506-bib-0003] Blascovich, J. (2008). Challenge and threat. In A. J. Elliot (Ed.), Handbook of approach and avoidance motivation (pp. 431–445). Psychology Press. 10.1016/S0065-2601(08)60235-X

[bjep12506-bib-0004] Cacioppo, J. T. , Tassinary, L. G. , & Berntson, G. G. (2017). Strong inference in psychophysiological science. In J. T. Cacioppo , L. G. Tassinary , & G. G. Berntson (Eds.), Handbook of psychophysiology, 4th ed. (pp. 3–15). Cambridge University Press.

[bjep12506-bib-0005] Chang, M.‐L. (2020). Emotion display rules, emotion regulation, and teacher burnout. Frontiers in Education, 5(90), 10.3389/feduc.2020.00090

[bjep12506-bib-0006] Donker, M. H. (2020). In DEPTh: Dynamics of emotional processes in teachers – An exploration of teachers’ interpersonal behavior and physiological responses. .

[bjep12506-bib-0007] Donker, M. H. , Van Gog, T. , Goetz, T. , Roos, A.‐L. , & Mainhard, T. (2020). Associations between teachers’ interpersonal behavior, physiological arousal, and lesson‐focused emotions. Contemporary Educational Psychology, 63, 101906. 10.1016/j.cedpsych.2020.101906

[bjep12506-bib-0008] Donker, M. H. , Van Gog, T. , & Mainhard, M. T. (2018). A quantitative exploration of two teachers with contrasting emotions: intra‐individual process analyses of physiology and interpersonal behavior. Frontline Learning Research, 6(3), 162–184. 10.14786/flr.v6i3.372

[bjep12506-bib-0009] Donker, M. H. , Van Vemde, L. , Hessen, D. J. , Van Gog, T. , & Mainhard, T. (2021). Observational, student, and teacher perspectives on interpersonal teacher behavior: shared and unique associations with teacher and student emotions. Learning and Instruction, 73, 101414. 10.1016/j.learninstruc.2020.101414

[bjep12506-bib-0010] Ebner‐Priemer, U. W. , & Kubiak, T. (2007). Psychological and psychophysiological ambulatory monitoring. European Journal of Psychological Assessment, 23(4), 214–226. 10.1027/1015-5759.23.4.214

[bjep12506-bib-0011] Fabrigar, L. R. , Visser, P. S. , & Browne, M. W. (1997). Conceptual and methodological issues in testing the circumplex structure of data in personality and social psychology. Personality and Social Psychology Review, 1(3), 184–203. 10.1207/s15327957pspr0103_1 15659349

[bjep12506-bib-0012] Frenzel, A. C. , Goetz, T. , Lüdtke, O. , Pekrun, R. , & Sutton, R. E. (2009). Emotional transmission in the classroom: Exploring the relationship between teacher and student enjoyment. Journal of Educational Psychology, 101(3), 705–716. 10.1037/a0014695

[bjep12506-bib-0013] Frenzel, A. C. , Pekrun, R. , & Goetz, T. (2007). Girls and mathematics —A “hopeless” issue? A control‐value approach to gender differences in emotions towards mathematics. European Journal of Psychology of Education, 22(4), 497–514. 10.1007/BF03173468

[bjep12506-bib-0014] Friedman, I. A. (2003). Self‐efficacy and burnout in teaching: The importance of interpersonal‐relations efficacy. Social Psychology of Education, 6(3), 191–215. 10.1023/A:1024723124467

[bjep12506-bib-0015] Goetz, T. , Bieleke, M. , Gogol, K. , van Tartwijk, J. , Mainhard, T. , Lipnevich, A. A. , & Pekrun, R. (2021). Getting along and feeling good: Reciprocal associations between student‐teacher relationship quality and students’ emotions. Learning and Instruction, 71, 101349. 10.1016/j.learninstruc.2020.101349.

[bjep12506-bib-0016] Goetz, T. , Cronjaeger, H. , Frenzel, A. C. , Lüdtke, O. , & Hall, N. C. (2010). Academic self‐concept and emotion relations: Domain specificity and age effects. Contemporary Educational Psychology, 35(1), 44–58. 10.1016/j.cedpsych.2009.10.001

[bjep12506-bib-0017] Goetz, T. , Lüdtke, O. , Nett, U. E. , Keller, M. M. , & Lipnevich, A. A. (2013). Characteristics of teaching and students’ emotions in the classroom: Investigating differences across domains. Contemporary Educational Psychology, 38(4), 383–394. 10.1016/j.cedpsych.2013.08.001

[bjep12506-bib-0018] Goetz, T. , Pekrun, R. , Hall, N. , & Haag, L. (2006). Academic emotions from a social‐cognitive perspective: Antecedents and domain specificity of students’ affect in the context of Latin instruction. British Journal of Educational Psychology, 76(2), 289–308. 10.1348/000709905X42860 16719965

[bjep12506-bib-0019] Gratton, G. , & Fabiani, M. (2017). Biosignal processing in psychophysiology: Principles and current developments. In J. T. Cacioppo , L. G. Tassinary , & G. G. Berntson (Eds.), Handbook of psychophysiology, 4th ed. (pp. 628–661). Cambridge University Press. 10.1117/1.NPh.4.3.031208

[bjep12506-bib-0020] Gross, J. J. (1998). Antecedent‐and response‐focused emotion regulation: Divergent consequences for experience, expression, and physiology. Journal of Personality and Social Psychology, 74(1), 224. 10.1037//0022-3514.74.1.224 9457784

[bjep12506-bib-0021] Horowitz, L. M. , & Strack, S. (2010). Handbook of interpersonal psychology: Theory, research, assessment, and therapeutic interventions. John Wiley & Sons. 10.1002/9781118001868

[bjep12506-bib-0022] Houtveen, J. H. , & Geus, E. J. C. (2009). Noninvasive psychophysiological ambulatory recordings. European Psychologist, 14(2), 132–141. 10.1027/1016-9040.14.2.132

[bjep12506-bib-0023] Johnston, D. , Bell, C. , Jones, M. , Farquharson, B. , Allan, J. , Schofield, P. , Ricketts, I. , & Johnston, M. (2015). Stressors, appraisal of stressors, experienced stress and cardiac response: A real‐time, real‐life investigation of work stress in nurses. Annals of Behavioral Medicine, 50(2), 187–197. 10.1007/s12160-015-9746-8 PMC4823345 26608281

[bjep12506-bib-0024] Junker, R. , Donker, M. H. , & Mainhard, T. (2021). Potential classroom stressors of teachers: An audiovisual and physiological approach. Learning and Instruction, 75, 101495. 10.1016/j.learninstruc.2021.101495.

[bjep12506-bib-0025] Keller, M. M. , & Becker, E. S. (2021). Teachers’ emotions and emotional authenticity: Do they matter to students’ emotional responses in the classroom? Teachers and Teaching, 27(5), 404–422. 10.1080/13540602.2020.1834380

[bjep12506-bib-0026] Keller, M. M. , Becker, E. S. , Frenzel, A. C. , & Taxer, J. L. (2018). When teacher enthusiasm is authentic or inauthentic: Lesson profiles of teacher enthusiasm and relations to students’ emotions. AERA Open, 4(4), 1–16. 10.1177/2332858418782967

[bjep12506-bib-0027] Koo, T. K. , & Li, M. Y. (2016). A guideline of selecting and reporting Intraclass Correlation Coefficients for reliability research. Journal of Chiropractic Medicine, 15, 155–163. 10.1016/j.jcm.2016.02.012 27330520 PMC4913118

[bjep12506-bib-0028] Kreibig, S. D. (2010). Autonomic nervous system activity in emotion: A review. Biological Psychology, 84(3), 394–421. 10.1016/j.biopsycho.2010.03.010 20371374

[bjep12506-bib-0029] Kunter, M. , Baumert, J. , & Köller, O. (2007). Effective classroom management and the development of subject‐related interest. Learning and Instruction, 17(5), 494–509. 10.1016/j.learninstruc.2007.09.002

[bjep12506-bib-0030] LeBreton, J. M. , & Senter, J. L. (2007). Answers to 20 questions about interrater reliability and interrater agreement. Organizational Research Methods, 11(4), 815–852. 10.1177/1094428106296642

[bjep12506-bib-0031] Lei, H. , Cui, Y. , & Chiu, M. M. (2018). The relationship between teacher support and students’ academic emotions: A meta‐analysis. Frontiers in Psychology, 8(2288), 10.3389/fpsyg.2017.02288 PMC5786576 29403405

[bjep12506-bib-0032] Lizdek, I. , Sadler, P. , Woody, E. , Ethier, N. , & Malet, G. (2012). Capturing the stream of behavior: A computer‐joystick method for coding interpersonal behavior continuously over time. Social Science Computer Review, 30(4), 513–521. 10.1177/0894439312436487

[bjep12506-bib-0033] Mainhard, T. , Oudman, S. , Hornstra, L. , Bosker, R. J. , & Goetz, T. (2018). Student emotions in class: The relative importance of teachers and their interpersonal relations with students. Learning and Instruction, 53, 109–119. 10.1016/j.learninstruc.2017.07.011

[bjep12506-bib-0034] Martin, A. J. , & Dowson, M. (2009). Interpersonal relationships, motivation, engagement, and achievement: Yields for theory, current issues, and educational practice. Review of Educational Research, 79(1), 327–365. 10.3102/0034654308325583

[bjep12506-bib-0035] Murray‐Close, D. , Holterman, L. A. , Breslend, N. L. , & Sullivan, A. (2017). Psychophysiology of proactive and reactive relational aggression. Biological Psychology, 130, 77–85. 10.1016/j.biopsycho.2017.10.005 29055714

[bjep12506-bib-0036] Myrtek, M. (2004). Heart and emotion: Ambulatory monitoring studies in everyday life. Hogrefe & Huber Publishers.

[bjep12506-bib-0037] Naim, R. , Goodwin, M. S. , Dombek, K. , Revzina, O. , Agorsor, C. , Lee, K. , Zapp, C. , Freitag, G. F. , Haller, S. P. , Cardinale, E. , Jangraw, D. , & Brotman, M. A. (2021). Cardiovascular reactivity as a measure of irritability in a transdiagnostic sample of youth: Preliminary associations. International Journal of Methods in Psychiatric Research, 30(4), e1890. 10.1002/mpr.1890 34390050 PMC8633925

[bjep12506-bib-0038] Parkinson, B. , Fischer, A. H. , & Manstead, A. S. (2005). Emotion in social relations: Cultural, group, and interpersonal processes. Psychology Press. 10.4324/9780203644966

[bjep12506-bib-0039] Pekrun, R. (2006). The control‐value theory of achievement emotions: Assumptions, corollaries, and implications for educational research and practice. Educational Psychology Review, 18(4), 315–341. 10.1007/s10648-006-9029-9

[bjep12506-bib-0040] Pekrun, R. (2017). Achievement emotions. In R. Patluny , A. Bellocchi , R. E. Olson , S. Khorana , & M. Peterie (Eds.), Emotions in late modernity (pp. 142–158). Routledge. 10.4324/9781351133319

[bjep12506-bib-0041] Pekrun, R. , Goetz, T. , Frenzel, A. C. , Barchfeld, P. , & Perry, R. P. (2011). Measuring emotions in students’ learning and performance: The Achievement Emotions Questionnaire (AEQ). Contemporary Educational Psychology, 36(1), 36–48. 10.1016/j.cedpsych.2010.10.002

[bjep12506-bib-0042] Porges, S. W. , & Byrne, E. A. (1992). Research methods for measurement of heart rate and respiration. Biological Psychology, 34(2), 93–130. 10.1016/0301-0511(92)90012-J 1467397

[bjep12506-bib-0043] Rafaeli, A. , & Sutton, R. I. (1989). The expression of emotion in organizational life. Research in Organizational Behavior, 11(1), 1–42. 10.4337/9781848443778.00032

[bjep12506-bib-0044] Rohrmann, S. , Bechtoldt, M. N. , Hopp, H. , Hodapp, V. , & Zapf, D. (2011). Psychophysiological effects of emotional display rules and the moderating role of trait anger in a simulated call center. Anxiety, Stress, & Coping, 24(4), 421–438. 10.1080/10615806.2010.530262 21077009

[bjep12506-bib-0045] Sadler, P. , Ethier, N. , Gunn, G. R. , Duong, D. , & Woody, E. (2009). Are we on the same wavelength? Interpersonal complementarity as shared cyclical patterns during interactions. Journal of Personality and Social Psychology, 97(6), 1005–1020. 10.1037/a0016232 19968416

[bjep12506-bib-0046] Scheepers, D. , De Wit, F. , Ellemers, N. , & Sassenberg, K. (2012). Social power makes the heart work more efficiently: evidence from cardiovascular markers of challenge and threat. Journal of Experimental Social Psychology, 48(1), 371–374. 10.1016/j.jesp.2011.06.014

[bjep12506-bib-0047] Scholl, A. , De Wit, F. , Ellemers, N. , Fetterman, A. K. , Sassenberg, K. , & Scheepers, D. (2018). The burden of power: construing power as responsibility (rather than as opportunity) alters threat‐challenge responses. Personality and Social Psychology Bulletin, 44(7), 1024–1038. 10.1177/0146167218757452 29544390

[bjep12506-bib-0048] Stark, K. , & Bettini, E. (2021). Teachers’ perceptions of emotional display rules in schools: A systematic review. Teaching and Teacher Education, 104, 103388. 10.1016/j.tate.2021.103388.

[bjep12506-bib-0049] Sun, X. , Hendrickx, M. M. H. G. , Goetz, T. , Wubbels, T. , & Mainhard, T. (2022). Classroom social environment as student emotions’ antecedent: Mediating role of achievement goals. The Journal of Experimental Education, 90(1), 146–157. 10.1080/00220973.2020.1724851

[bjep12506-bib-0050] Taxer, J. L. , & Frenzel, A. C. (2015). Facets of teachers’ emotional lives: A quantitative investigation of teachers’ genuine, faked, and hidden emotions. Teaching and Teacher Education, 49, 78–88. 10.1016/j.tate.2015.03.003

[bjep12506-bib-0051] Taxer, J. L. , & Frenzel, A. C. (2018). Inauthentic expressions of enthusiasm: Exploring the cost of emotional dissonance in teachers. Learning and Instruction, 53, 74–88. 10.1016/j.learninstruc.2017.07.008

[bjep12506-bib-0052] Van Dijk, A. E. , Van Lien, R. , Van Eijsden, M. , Gemke, R. J. B. J. , Vrijkotte, T. G. M. , & De Geus, E. J. (2013). Measuring cardiac Autonomic Nervous System (ANS) activity in children. Journal of Visualized Experiments, (74), 10.3791/50073 PMC3667644 23666435

[bjep12506-bib-0053] Van Kleef, G. A. , & Côté, S. (2022). The social effects of emotions. Annual Review of Psychology, 73(1), 629–658. 10.1146/annurev-psych-020821-010855 34280326

[bjep12506-bib-0054] Vrijkotte, T. G. M. , Van Doornen, L. J. P. , & De Geus, E. J. C. (2000). Effects of work stress on ambulatory blood pressure, heart rate, and heart rate variability. Hypertension, 35(4), 880–886. 10.1161/01.hyp.35.4.880 10775555

[bjep12506-bib-0056] Watson, D. , & Tellegen, A. (1985). Toward a consensual structure of mood. Psychological Bulletin, 98(2), 219–235. 10.1037/0033-2909.98.2.219 3901060

[bjep12506-bib-0057] Wentzel, K. R. , & Ramani, G. B. (2016). Handbook of social influences in school contexts: Social‐emotional, motivation, and cognitive outcomes. Routledge. 10.4324/9781315769929

[bjep12506-bib-0058] Willemsen, G. H. , De Geus, E. J. , Klaver, C. H. , Van Doornen, L. J. , & Carrofl, D. (1996). Ambulatory monitoring of the impedance cardiogram. Psychophysiology, 33, 184–193. 10.1111/j.1469-8986.1996.tb02122.x 8851246

[bjep12506-bib-0059] Wubbels, T. , Créton, H. A. , & Hooymayers, H. P. Discipline problems of beginning teachers, interactional teacher behaviour mapped out. Abstracted in resources in education, 20(12), 153. ERIC document ED260040.

[bjep12506-bib-0060] Xanthopoulou, D. , Bakker, A. B. , Oerlemans, W. G. M. , & Koszucka, M. (2018). Need for recovery after emotional labor: Differential effects of daily deep and surface acting. Journal of Organizational Behavior, 39(4), 481–494. 10.1002/job.2245

